# Impact of solid waste disposal on heavy metals concentration and distribution in a naturally remediated landfill site

**DOI:** 10.1007/s10661-026-15304-2

**Published:** 2026-04-17

**Authors:** Celuxolo Michal Dlamini, Sizwe Thamsanqa Hlatshwayo, Nontokozo Pertunia Mkhonza

**Affiliations:** 1 Discipline of Geography, School of Agriculture and Science, University of KwaZulu-Natal, Bag X01, Pietermaritzburg, Scottsville, 3209 South Africa; 2 Discipline of Agricultural Science, School of Agriculture and Science, University of KwaZulu-Natal, Pietermaritzburg Campus, P.O. Bag X01, Scottsville, 3209 South Africa

**Keywords:** Dumping sites, Environmental health risk, Toxic elements, Trace elements, Waste management

## Abstract

South Africa produces approximately 122 million tons of solid waste annually, which poses a significant environmental concern. The decomposition of solid waste in landfill sites results in the release of toxic heavy metals into the soil, posing a threat to human health and biodiversity. This study evaluated the impact of solid waste disposal in the distribution of heavy metals, that is, lead (Pb), zinc (Zn), cadmium (Cd), and arsenic (As), in landfill site soils. Soil samples collected (0-20 cm depth) from five dumping cells, two non-dumping zones, and a wetland were analyzed for soil chemical properties (exchangeable bases, extractable phosphorus (P), and micronutrients) and heavy metal concentrations. The results showed clear spatial variation in nutrient concentration across the landfill, with boundaries exhibiting lower fertility than dumping cells. The results indicated that cell 5 had higher organic matter concentration compared to all the treatments, except for cell 4. Heavy metal concentration results showed that Cd was below detection (< 1 mg kg^−1^) in the boundaries, wetlands, and cell 5 but exceeded the permissible levels (17.5-32.8 mg kg^−1^) in cells 1 to 4. Pb exceeded the permissible limit (50 mg kg^−1^) across all zones, with cell 5 showing concentrations almost double the ones in boundaries and wetland. Zinc concentrations were extremely high in cell 5 (1795 mg kg^−1^), > 100% higher than other treatments, while As remained relatively uniform across the treatments. These findings demonstrate the importance of natural remediation in reducing contaminant mobility and provide landfill sites management insights.

## Introduction

South Africa generates more than 122 million tons of solid waste annually, mainly due to high population density in major cities (Dlamini et al., 2019). Given this swift escalation of solid waste generation in South Africa, coupled with a rising population, waste management is anticipated to reach an irreversible situation (Mavropoulos & Nilsen, 2020). South Africa, restricted by limited resources due to financial constraints, encounters a challenge in coping with the escalating population and struggles to effectively manage the resulting waste generation (Akpan & Olukanni, 2020; Viljoen et al., 2021). Kubanza and Simatele (2020) reported that major cities in South Africa are struggling to implement integrated waste management due to excessive waste production in concentrated urban areas. In South Africa, waste minimization through recycling and reusing is not prevalent, primarily due to lack of awareness regarding its significance and societal perceptions of waste (Godfrey, 2021) . In addition, informal waste recycling and sorting activities are often regarded as degrading jobs in this country. The adoption of recycling is primarily observed in the private sector, where profitability is derived from the resale of recyclable products (Ghisellini & Ulgiati, 2020). Consequently, South Africa remains with limited alternatives for sustainable waste management, leading to a heavy reliance on landfill sites for waste disposal.

Anthropogenic activities, including but not limited to painting, disposal of electronic waste, municipal waste, aged metals, pesticide and herbicide usage, fertilizer application, vehicle oil emissions, and mining, contribute to the introduction of heavy metals into the environment (Alloway, 2012). Heavy metals occur naturally in the earth’s crust and are typically categorized in the periodic table of elements, exhibiting characteristic metallic properties (Ali & Khan, 2018). As solid waste decomposes in landfill sites, the release of heavy metals occurs, where increasing concentrations can potentially migrate beyond the landfill sites, contaminating surrounding soils, including agricultural lands (Lee et al., 2016; Samadder et al., 2017). Moreover, there is a potential risk of leaching into groundwater, posing threats to both the aquatic environment and humans relying on groundwater sources (Mishra et al., 2019). These heavy metals pose a significant environmental crisis due to their toxicity to both human and environmental health and their persistence in soils, because they degrade uneasily (Alloway, 2013; Friedlova, 2010; Hossain et al., 2011). Typically, landfill soils play a crucial role in the disposal of solid waste, serving as a filter that prevents leaching of decomposed waste chemicals into groundwater (Liu et al., 2019; Vodyanitskii, 2016). Therefore, sustainable construction of landfill sites is essential to safeguard the environment against pollution resulting from the decomposition of waste (Yuan, 2013). In this regard, it is imperative to construct landfill sites with measures aimed at minimizing heavy metal leaching.

Despite the extensive research on heavy metal contamination in active landfills, critical gaps persist in understanding the long-term behavior of heavy metals in naturally remediated landfill sites, particularly in South Africa. The effectiveness of the phytoremediation process, microbial degradation, and soil buffer zones in reducing heavy metal concentration remains poorly quantified, especially in regions with acidic soils and high precipitation levels, which may accelerate heavy metal mobility and bioavailability (Dlamini et al., 2019). Soils of KwaZulu-Natal are highly acidic, and this may escalate heavy metal contamination through increased mobility (Kubanza & Simatele, 2020). In addition, the role of buffer zones in reducing heavy metal migration has not been validated for non-engineered landfills, which dominate waste disposal in developing countries, including South Africa. While studies from temperate climates suggest gradual metal immobilization reducing solubility and bioavailability over time (Samadder et al., 2017), the kinetics of this process in subtropical African environments where warmer temperatures and intense weathering may alter metal speciation are poorly understood.

Extensive research findings highlight the influence of landfill boundaries on heavy metal migration and accumulation in adjacent soils and vegetation, with evidence of contamination extending beyond landfill boundaries (Azimov et al., 2020; Makuleke and Ngole-Jeme, 2020; Alao, 2025). Several studies observed that landfill site boundaries act as partial barriers influencing the spatial distribution and migration of heavy metals, often showing contamination decreases beyond landfill perimeters (Azimov et al., 2020; Bahaa-eldin et al., 2011; He et al., 2022). Conversely, some research findings demonstrate continuous lateral migration of metals beyond boundaries, with notable heavy metal presence in adjacent soils or water bodies, suggesting leachate leakage (Gyabaah et al., 2023; Vongdala et al., 2019; Ashraf et al., 2013). The inconsistency in observation could be due to the fact that heavy metal mobility is related to soil pH, organic matter content, and slope position which differ from different landfills. Therefore, this study aims to assess the impact of solid waste disposal on heavy metal concentrations and distribution in a naturally remediated landfill site. The specific objectives are to (i) quantify the total concentration of Pb, Zn, Cd, and As across different landfill zones; (ii) evaluate the effectiveness of the landfill boundaries as a buffer zone in reducing heavy metal migration; and (iii) analyze the role of phytoremediated zones in reducing heavy metal concentration.

## Materials and methods

### Study site and experimental design

**Fig. 1:**
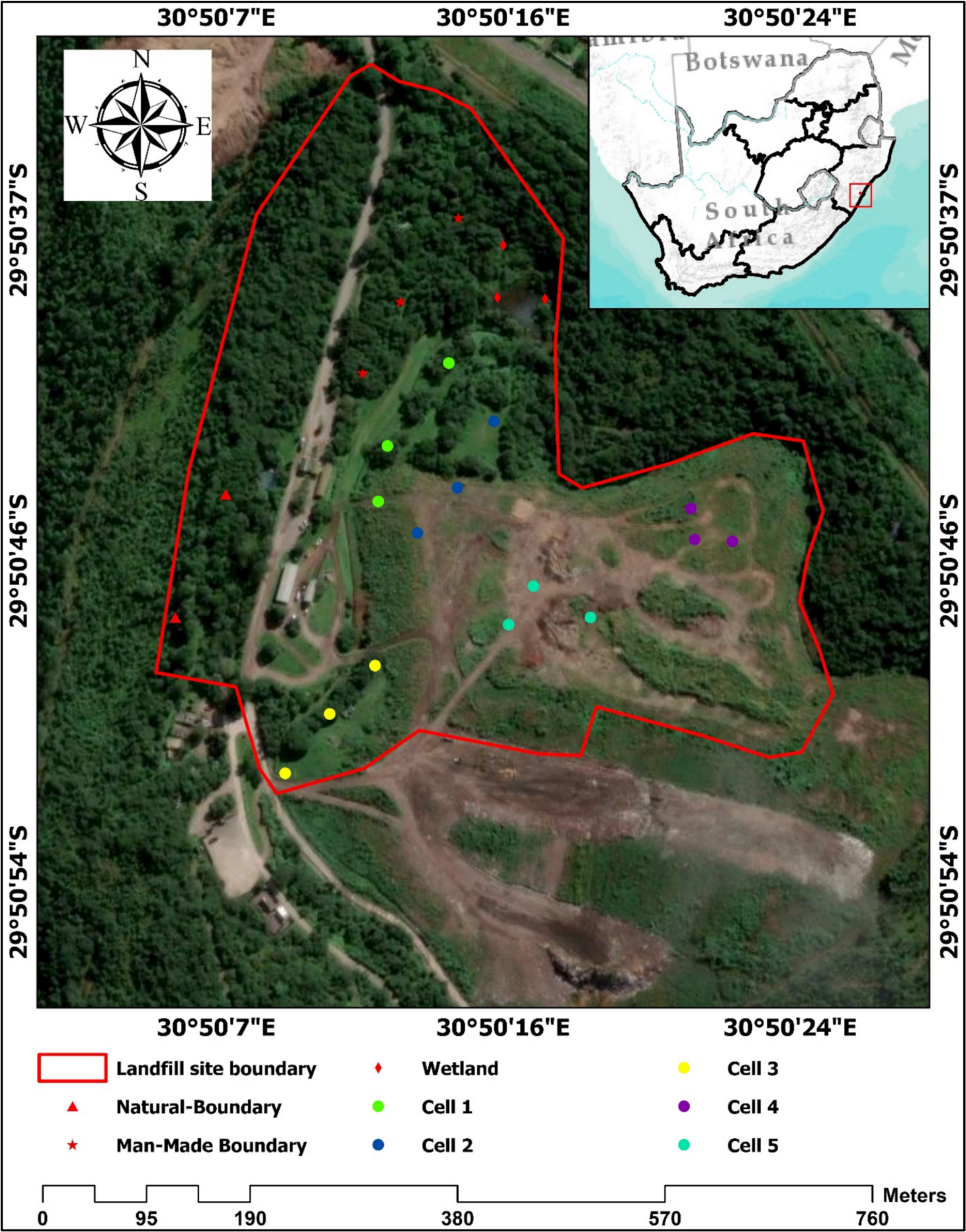
Experimental site location with sample points of each sampling zone in the landfill site

The study was conducted at the Mariannhill landfill site (latitude -29.85 and longitude 30.84) in Pinetown, South Africa (Fig. 1). The eThekwini Municipality and Durban Solid Waste (DSW) initiated the landfill site in 1997 and had it registered as G: L: B+ (Large General Site) of South Africa (Zulu, 2018). The landfill site has a total area covering 49.5 ha. The landfill site is elevated at 200 m above sea level and receives 545 mm average rainfall annually. The area records an average air temperature of 19.6℃, ranging from a minimum of 13.2℃ to a maximum of 26.6℃ per year. The landfill site receives at least 450 tons of solid waste per day, including but not limited to household, municipal, industrial, and agricultural waste. The landfill incorporates a rotation system with five dumping cells, using one cell at a time for waste disposal. Once a cell reaches full capacity, it undergoes rehabilitation through tree planting, soil utilization, and compaction to restore surrounding and native biodiversity. The landfill site consists of five waste dumping cells, a wetland, a natural boundary, a man-made boundary, a conservancy area, and a gas-electricity plant (Parkin et al., 2006). The man-made boundary of the landfill site is the main source of the soil used for waste compaction and replanting of trees in the full capacity cells (Mbazima et al., 2022). This sequential rehabilitation process ensures that a new cell is created after the completion of each rehabilitation phase (Sawyerr et al., 2021). The landfill’s wetland functions as a natural filter for leachate drained from rainfall (Sawyerr et al., 2021). The water passes through the dumping cells, ultimately reaching the wetland where thorough filtration takes place (Dell’orto & Trois, 2024). The filtration process involves the use of sand at the water exit points of the wetland and clay soils within the wetland itself, acting as a filter. Cells 1 and 2 were fully rehabilitated, while cell 3 was ¾ rehabilitated. Cells 4 and 5 were active dumping zones. Moreover, each cell is equipped with a network of gas-collecting pipes beneath, channeling the collected gas to a dedicated gas-electricity plant for electricity generation. No waste has been deposited in the natural boundary, which functions as a buffer zone for the landfill site. Furthermore, the man-made boundary has been constructed within the natural boundary to enhance the resilience of the natural buffer zone. These man-made and natural boundaries are used for the conservation of various plant (i.e., *Acacia ataxacantha*, *Acokanthera oppositifolia*) and animal (*Apalis thoracica*, *Trachyphonus vaillantii*) species that were relocated from the dumping zone.

### Soil sampling

**Fig. 2:**
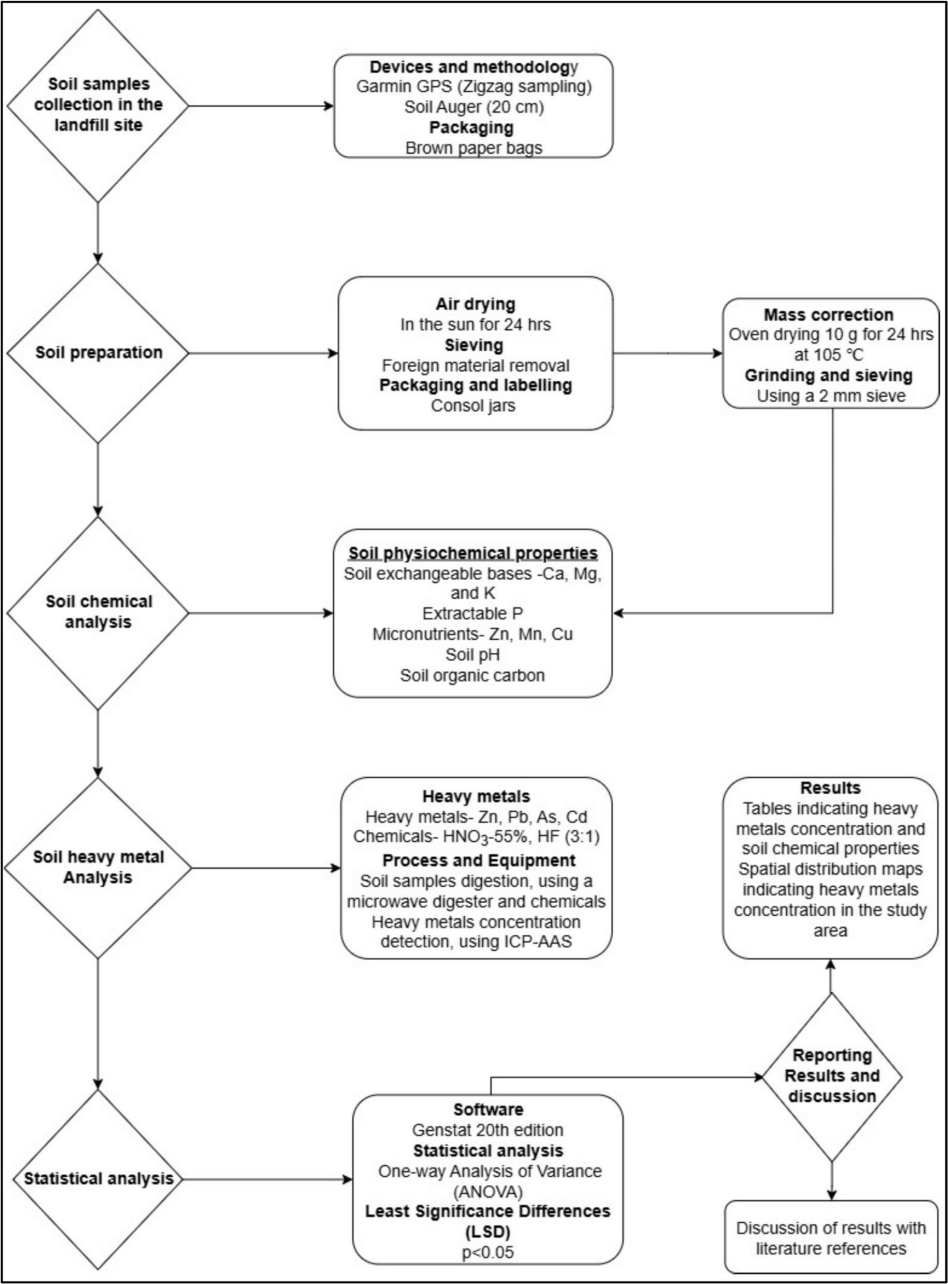
Flow diagram presenting methodology for soil data collection, analysis, and presentation of results

The soil samples were collected in August 2022, during the winter season of the year using the zigzag method in each cell. The soil samples were collected in the top 20 cm depth using soil augers (20 cm × 3.5 cm) from eight sites (natural boundary, man-made boundary, cells 1–5, and wetland) within the landfill site (Fig. 2). Geographic Positioning System (GPS) coordinates for each sampling point were recorded using a Garmin eTrex 10 handheld GPS device. To determine whether contaminants are transported during rainfall via surface runoff, soils were sampled from the wetland. To capture variability, three replicates were collected from each sampling site (Adhikari et al., 2024), resulting in a total of 24 samples. For each replicate, four subsamples were collected. Due to safety concerns in wetlands, three soil replicates were collected along the perimeter, representing all water entry locations of the wetland. The natural boundary and man-made boundaries were sampled as control samples to determine the effectiveness of the remediation methods used in the landfill site. Prior to air-drying the samples, soil moisture was determined by measuring approximately 10 g of each soil sample using a mass scale (Marschner & Zheng, 2022). Thereafter, the soil samples were oven dried at 105℃ overnight to determine the soil moisture content of the soil using initial and final mass (Allende-Montalbán et al., 2024). The soils were mixed, air-dried, and homogenized for each replicate to make a composite sample. Thereafter, the soil samples were sieved through a 2 mm sieve before laboratory analysis.

### Soil chemical analysis and organic matter

Soil pH, exchangeable bases, and acidity were analyzed using methods documented by Manson et al. (2020) (Fig. 2). Briefly, soil pH was analyzed in 1 M KCl (1:2.5 soil to KCl suspension). Exchangeable bases (Ca^2+^ and Mg^2+^) and acidity were extracted using a 25 mL 1 M KCl solution after shaking for 10 min in a shaker at a speed of 400 r.p.m. After that, atomic absorption spectrophotometry (Varian 2600) was used to determine the concentration of Ca^2+^ and Mg^2+^. From the same extract, exchangeable acidity was determined using acid–base titration. Potassium, Zn, Mn, and Cu were extracted using the Ambic-2 method, followed by quantification using atomic adsorption spectrophotometry (Varian 2600) (Manson et al. 2020). Soil organic carbon was determined using the Walkley Black method (Walkley & Black, 1934). Thereafter, organic matter content was determined using the conversion factor of 1.72.

### Heavy metals analysis

The extraction of soil heavy metals (Fig. 2) was conducted using a highly concentrated nitric acid (HNO_3_-55%) and hydrofluoric acid (HF-40%) mixture at a volume ratio of 3:1 (HNO_3_:HF). The soil samples were digested using the Anton Paar Multiwave 5000 microwave digester, as detailed by Turek et al. (2019), and the concentration of heavy metals was determined using the ICP-AAS Varian 720-ES ICP-AES (Varian, California, USA).

### Statistical analysis

The soil fertility parameters and heavy metal concentration were analyzed using the one-way analysis of variance (ANOVA) using GenStat 20th edition (Payne et al., 2012). The difference between the normally distributed treatment means was determined using least significant differences (LSD) at a significance level of *p* < 0.05.

## Results and discussion

### Soil chemical properties and organic matter

**Fig. 3:**
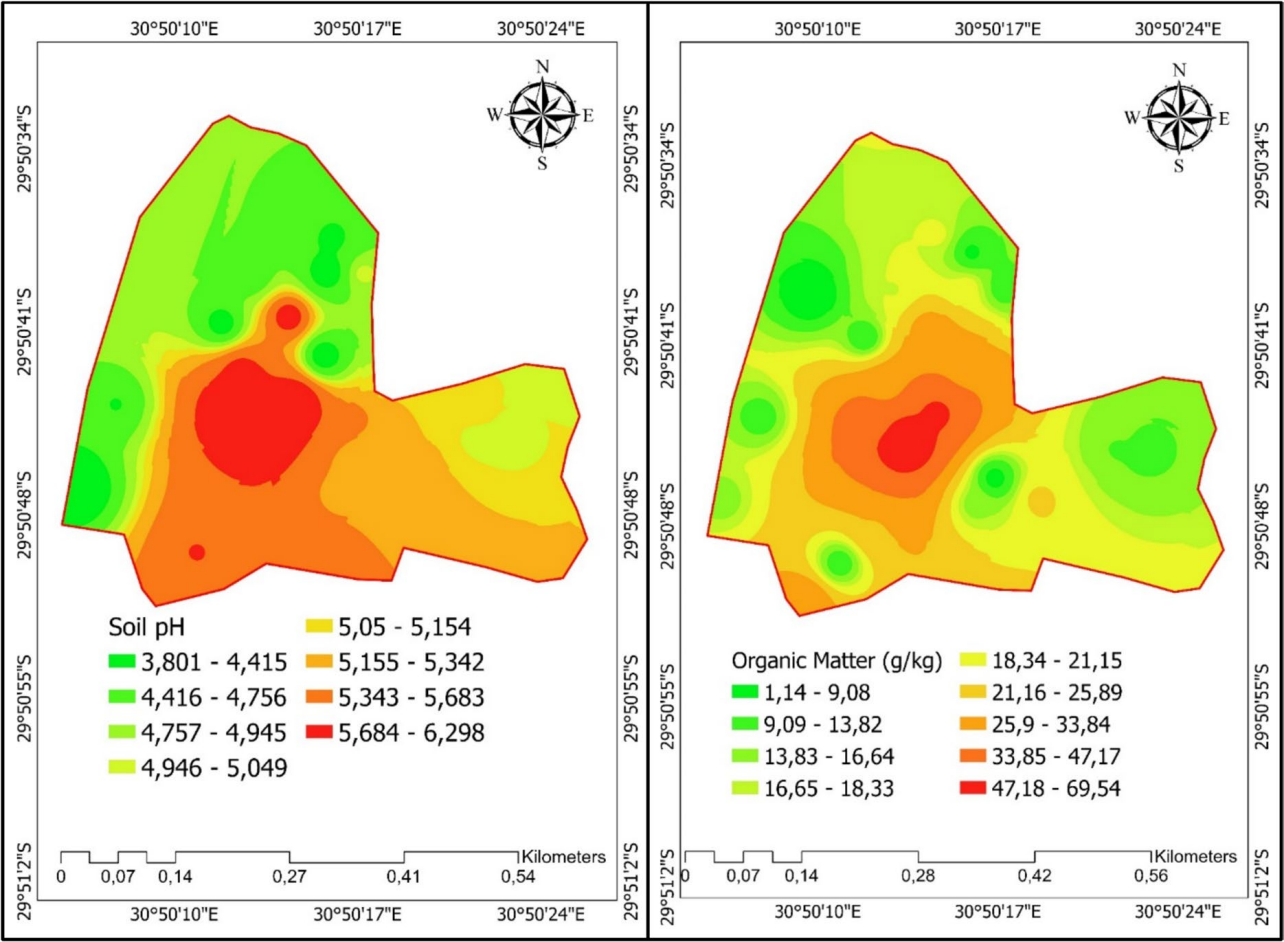
Spatial distribution map indicating soil pH and organic matter content across the sampling zones in the landfill site

**Fig. 4:**
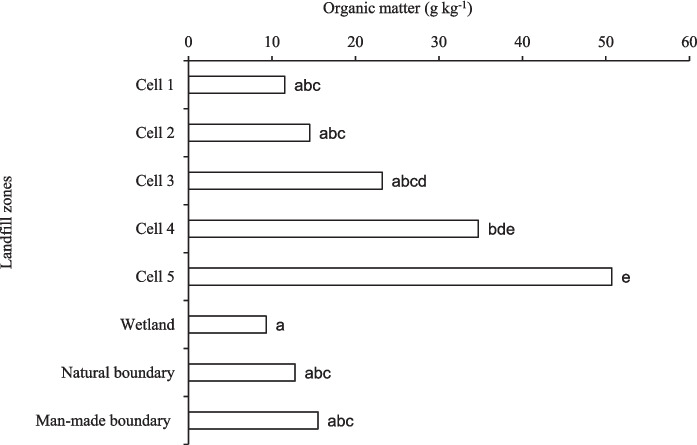
Soil organic matter content in different landfill zones

Soil organic matter varied significantly (*p* = 0.003) among the treatments, with cell 5 exhibiting higher organic matter content (50.7 g kg^−1^) than all treatments, except for cell 4 (34.7 g kg^−1^), which was comparable to cell 5 (Table 1, Figs. 3 and 4). The significant variations in soil organic matter (SOM) across landfill zones indicate both the history of waste input and the stage of rehabilitation. The significantly higher SOM content in active dumping cells aligns with the continuous accumulation of fresh organic-rich waste (e.g., paper, garden residue, and food waste), which results in labile carbon pools that accumulate prior to mineralization (Esiana, 2015). Higher levels of organic matter in landfill soils are typically associated with higher levels of carbon because of waste that is partially decomposed. Conversely, lower SOM in rehabilitated cells, boundaries, and wetland soils signifies decomposition and stabilization linked to vegetative cover and soil development (Jensen et al., 2020). This pattern corresponds with findings that improved vegetation and soil cover on rehabilitated landfill surfaces promote organic matter turnover via microbial processing and aggregation, resulting in more stable carbon pools relative to active disposal sites.

**Table 1:** Selected soil extractable macro- and micronutrients, and soil pH

Treatment	P	K	Ca	Mg	Soil pH	Zn	Mn	Cu
	mg kg^−1^							
Cell 1	9 ± 3.5a	110 ± 31.2a	802 ± 289.3a	183 ± 24.3ab	5.03 ± 0.1bc	3.1 ± 1.2a	35.6 ± 3.5	2.03 ± 1.1a
Cell 2	13 ± 2.5a	137 ± 54.2a	839 ± 219.7a	148 ± 30.7a	5.27 ± 0.1 cd	4.6 ± 1.3a	21.2 ± 3.9	4.54 ± 1.7ab
Cell 3	10 ± 3.1a	45 ± 7.5a	1008 ± 214.9a	154 ± 55a	5.57 ± 0.2cde	5.5 ± 3.1ab	23.0 ± 6.9	17.4 ± 3.3bc
Cell 4	31 ± 7.5c	467 ± 182.7b	2170 ± 257.5b	310 ± 39.1ab	5.87 ± 0.2de	10.9 ± 1.1bc	38.1 ± 24.1	27.6 ± 12.3c
Cell 5	28 ± 5.5bc	466 ± 134b	649 ± 201.1a	342 ± 106.1b	6.20 ± 0.1e	15.4 ± 3.3c	39.9 ± 11.4	21.7 ± 5.6c
Man-made boundary	18 ± 4.5ab	52 ± 10.6a	493 ± 148.7a	144 ± 24a	4.30 ± 0.5ab	4.9 ± 1.7a	57.7 ± 36.5	4.96 ± 1.6ab
Natural boundary	14 ± 2.3a	48 ± 8.5a	511 ± 275.1a	168 ± 55.5a	4.27 ± 0.2a	3.8 ± 0.6a	60.9 ± 29.8	3.82 ± 0.6ab
Wetland	10 ± 3.5a	51 ± 14.6a	406 ± 113.5a	249 ± 89.5ab	4.47 ± 0.5ab	3.9 ± 1.2a	40.6 ± 17.3	3.42 ± 1.0ab
*p* value	< 0.001	< 0.001	< 0.001	0.004	< 0.001	< 0.001	0.248	< 0.001
LSD	7.4	144.6	384	104	0.449	3.31	35.2	8.71

Different lowercase letters within a column indicate significant differences among means at *p* < 0.05 based on LSD; means sharing a letter are not significantly different. LSD represents the least significant difference

Notably, cell 4 exhibited the highest levels of P, K, Ca, and Cu at 31, 467, 2170, and 27.6 mg kg^−1^, indicating higher fertility compared to other treatments (Table 1). Cell 5 showed relatively higher Mg and Zn concentrations at 342 and 15.4 mg kg^−1^, respectively. Soil pH was highest in cell 5 at 6.2, indicating more neutral soil conditions in this treatment (Fig. 3). Mn levels, averaging at 39.9 mg kg^−1^ in cell 5, did not significantly differ across treatments (*p* = 0.248), indicating its uniform distribution across the landfill site. The natural boundary showed lower nutrient concentrations, with P, K, and Ca concentrations at 14, 48, and 511 mg kg^−1^, respectively, indicating less fertility compared to cells. The man-made boundary had similar nutrient levels to the natural boundary, with P, K, and Ca at 18, 52, and 493 mg kg^−1^, respectively, while the wetland displayed P, K, and Ca at 10, 51, and 406 mg kg^−1^, respectively, further indicating that these boundaries have lower nutrient availability compared to the cells. Nutrient enrichment in active cells, particularly increased concentration of P, K, and Ca, probably comes from direct waste inputs and ash residues (Ahmed et al., 2019). Alkaline wastes and Ca-rich materials that buffer acidity can raise the pH in some cells. This has been seen in landfill soil surveys where high organic loads and alkaline inputs keep conditions close to neutral to slightly alkaline.

### Heavy metals distribution

**Fig. 5:**
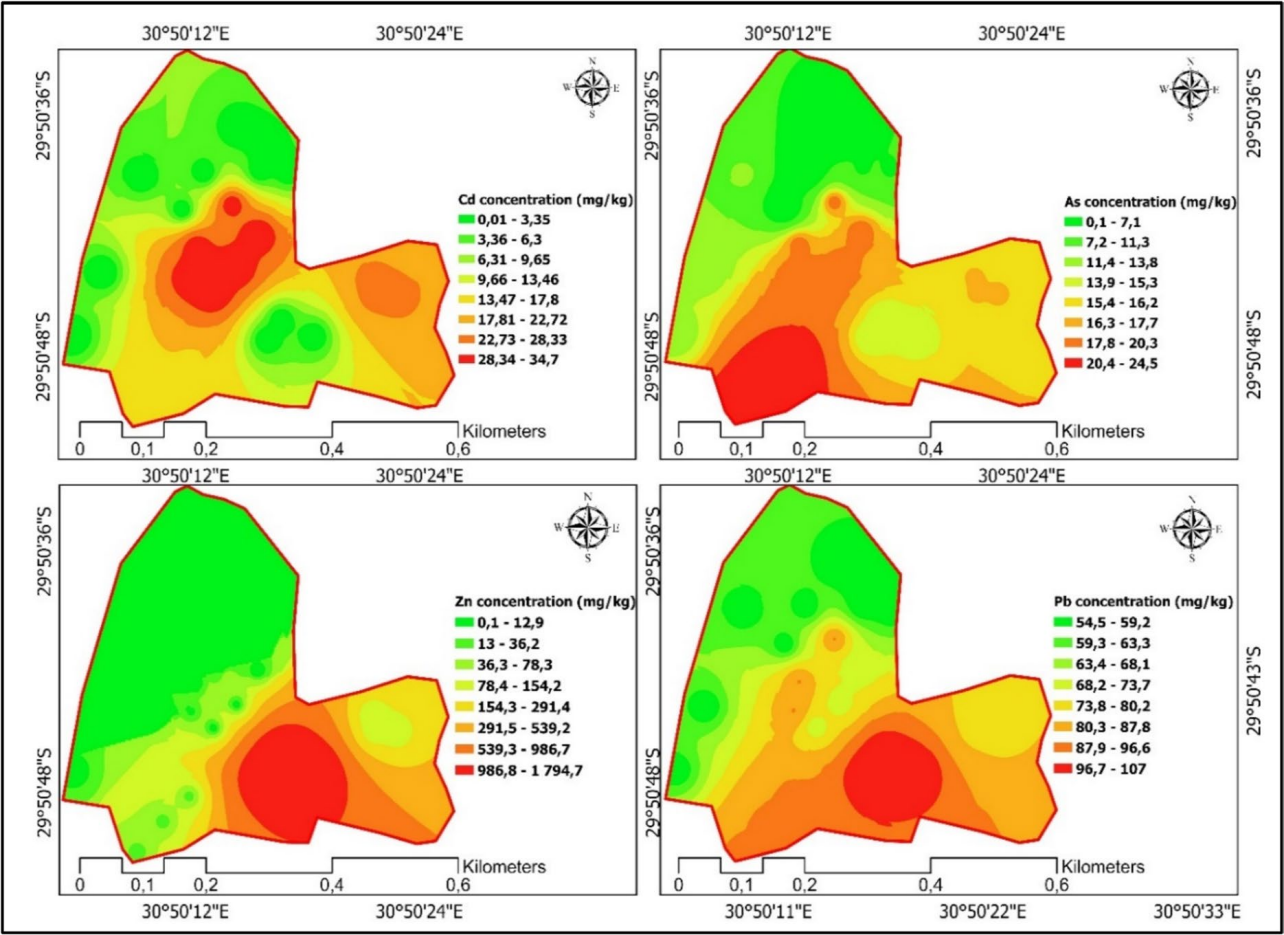
Spatial distribution maps indicating each heavy metal concentration across the sampling zones in the landfill site

The distribution of total heavy metals across the cells, natural boundaries, man-made boundary, and wetland (Table 2 and Fig. 5) could be explained by both waste input history and the effect of soil properties governing heavy metal mobility, solubility, retention, and stabilization in soils. The high Cd concentrations in cells 1 (average 32.8 mg kg^−1^) and 2 (average 34.7 mg kg⁻^1^) compared to other landfill zones indicate an inheritance effect of older waste inputs, particularly from historical deposition of Cd-rich materials such as batteries, plastics, and fertilizers (Agbeshie et al., 2020; Gebre & Debelie, 2015). In addition, Cd could have strongly sorbed to negatively charged clay edges and Fe/Al (hydr)oxides at the moderately acidic pH (5.0–5.3) observed in these cells, as well as the formation of stable inner-sphere complexes that increase persistence over time (Qin, 2024). Furthermore, rehabilitation with vegetation has the potential to add organic matter providing a binding site for Cd through carboxyl and phenolic functional groups, further immobilizing Cd, hence increasing total concentration (Liu et al., 2019). Moreover, soil compaction could create anaerobic microsites which could potentially promote Cd precipitation as cadmium sulfide (CdS) or carbonate (CdCO_3_), increasing the total Cd retention but reduce its bioavailability in the soil solution. Similar results of Cd accumulation in older landfill cells have been reported by Twumasi et al. (2016) in Ghanaian dumpsites and Kolawole et al. (2023) in Nigeria, suggesting that Cd persistence is a widespread problem in rehabilitated waste sites.

The concentrations of Pb exceeded the permissible limit (50 mg kg^−1^) across all treatments, with the highest concentrations recorded in cell 5 (107 mg kg^−1^) (Table 2). The observed Pb contamination could be linked to waste such as electronic waste, batteries, and paints (Pascale et al., 2016), which continue to be disposed of in active landfills (cells 5 and 4). The high Pb concentrations in cell 5 reflect recent inputs which may have high concentration of Pd, while the lower concentrations of Pb in rehabilitated cells suggest partial natural remediation through phytoremediation. However, lateral migration of Pb downslope into cell 4 likely occurred via leachate movement, consistent with observations by Chifamba (2007), who noted Pb transport with leachates along slope in landfill soils in Zimbabwe. The persistence of Pb in rehabilitated cells is similar to the findings of Makuleke and Ngole-Jeme (2020), who reported long-term Pb contamination in closed landfills despite natural remediation. Zinc (Zn) concentrations were significantly higher in cell 5 (1795 mg kg^−1^) compared to all the other treatments, far surpassing the permissible limit of 200 mg kg^−1^, and more than double the concentrations recorded in other cells. This reflects the continued deposition of Zn-rich waste such as galvanized metals, plastics, and batteries. Unlike Pb, Zn is less strongly sorbed to clays and oxides, particularly under acidic conditions. However, the relatively higher pH (6.2) and elevated organic matter (50.7 g kg⁻^1^) in cell 5 may enhance Zn retention through organic matter complexation and coprecipitation with carbonates. Despite this, Zn remains relatively mobile compared to Cd and Pb and thus represents a greater risk of leaching from active landfill cells. Similar observations of high Zn concentrations in active landfill soils have been documented by Nunes et al. (2014) in Mediterranean landfill environments and Alam et al. (2020) in Bangladesh, highlighting its mobility and ecological risk.

Arsenic (As) concentrations were relatively uniform across treatments (14.3–24.5 mg kg⁻^1^), with only cell 3 slightly exceeding the permissible threshold (20 mg kg^−1^) according to FAO/WHO standards. This uniformity suggests diffuse sources, such as residual inputs from historic pesticide applications or natural background levels derived from parent material. In acidic to neutral soils (pH 4.3–6.2), arsenate (AsO_4_^3-^) is the dominant form, which strongly adsorbs to Fe/Al oxides, limiting mobility. Although reducing conditions can convert arsenate to the more mobile arsenite (AsO_4_^3−^), the relatively stable distribution of As across landfill zones suggests effective buffering against redox fluctuations. Similar findings were reported by Chen et al. (2002) in Florida surface soils and Samadder et al. (2017) in Indian landfill soils, where As showed limited mobility compared to other heavy metals. The negligible heavy metal concentrations detected in the wetland, man-made boundary, and natural boundary, except for Pb, highlight their critical filtering capacity. These buffer zones function through adsorption onto clays and oxides, ion exchange, and co-precipitation processes that immobilize metals during leachate passage. Wetlands, in particular, provide redox gradients that promote Cd precipitation as CdS and facilitate the adsorption of Pb and As to oxide-rich sediments. The observed results are similar to the findings of Kumar et al. (2021), who demonstrated the role of phytoremediation and soil capping in reducing heavy metal mobility, and Bahaa-eldin et al. (2011), who reported boundary zones limiting heavy metal migration in Egyptian landfill soils. The wetlands and buffer zones are effective in mitigating migration, highlighting their importance in landfill design and management. These results emphasize the need for long-term monitoring of heavy metals in rehabilitated landfill sites, particularly focusing on bioavailable fractions, as also recommended by Moreno et al. (2009) and Wang and Hu (2023).

**Table 2:** Heavy metals concentrations and target values in soils (mg kg^−1^)

Treatment	Cd	Pb	As	Zn
Cell 1	32.8 ± 11.7c	88.0 ± 3.4ab	18.9 ± 11.6	7.50 ± 9.2a
Cell 2	34.7 ± 1.5c	69.9 ± 10.1a	19.5 ± 11	0.500 ± 0.7a
Cell 3	17.5 ± 14.4b	89.0 ± 45.8ab	24.5 ± 16.4	23.6 ± 21.1a
Cell 4	24.3 ± 8.6bc	78.4 ± 52.4ab	16.3 ± 15.1	92.6 ± 53.8a
Cell 5	0.00 ± 0a	107 ± 18.4b	14.3 ± 24.3	1795 ± 427.93b
Man-made boundary	0.00 ± 0a	55.9 ± 1.3a	0.00 ± 0	0.00 ± 0a
Natural boundary	0.00 ± 0a	56.2 ± 2.1a	11.9 ± 20.6	0.00 ± 0a
Wetland	0.00 ± 0a	54.4 ± 4.5a	6.69 ± 11.6	0.00 ± 0a
*p* value	< 0.001	0.028	0.696	< 0.001
LSD	13	33.2	29.6	106

Different lowercase letters within a column indicate significant differences among means at *p* < 0.05 based on LSD; means sharing a letter are not significantly different. LSD represents the least significant difference. Targets of permissible concentration of Cd = 1 mg kg^−1^; Pb = 50 mg kg^−1^; As = 20; and Zn = 200 mg kg^−1^ according to WHO/FAO (https://faolex.fao.org/docs/pdf/est97999E.pdf) standards

### Relationship between soil physicochemical properties, nutrients, and heavy metal content

The distribution of heavy metals across the landfill was strongly controlled by soil physicochemical properties and nutrient status. Higher soil pH and organic matter content in active dumping cells, particularly cell 5, promoted the retention of Pb and Zn through adsorption, organic complexation, and precipitation processes, resulting in elevated total heavy metal concentrations (Luo et al., 2024). In contrast, the acidic conditions and lower organic matter in wetland soils retained heavy metals’ mobility and limited their accumulation in the boundaries (Sun et al., 2021). Elevated Ca and Mg concentrations in active cells likely reduced Cd mobility through competitive sorption and carbonate formation, while higher phosphorus levels may have contributed to Pb immobilization via phosphate precipitation (Liao et al., 2023). Trace elements such as Zn, Mn, and Cu exhibited similar spatial trends to heavy metals, indicating shared geochemical controls and adsorption onto clay minerals and Fe/Mn (hydr)oxides (Kypritidou & Argyaraki, 2021). As showed relatively uniform concentrations across treatments, indicating strong adsorption to oxide-rich soil phases under acidic to near-neutral pH conditions. Overall, interactions among soil pH, organic matter, macronutrients, and trace elements collectively control the mobility, retention, and persistence of heavy metals within the landfill site.

## Conclusion

The findings revealed significant variation in heavy metal concentrations across different dumping zones, with some treatments exceeding the permissible limits in soils, indicating potential soil and groundwater contamination. The presence of these heavy metals highlights the risk associated with unmanaged landfill sites. In addition, these findings demonstrated that buffer zones and phytoremediation vary in their effectiveness in remediating heavy metal concentrations. The persistence of Pb in remediated cells suggests that there is a need for long-term monitoring and evaluation of heavy metal concentration in remediated dumpsites. Future research should investigate the bioavailable heavy metal pool in this landfill. These findings emphasize the urgent need for South Africa to adopt sustainable waste management strategies.
